# Incentives to promote accessing HIV care and viral suppression among HIV self-screening test users who obtain a reactive result

**DOI:** 10.3389/frph.2022.976021

**Published:** 2022-10-03

**Authors:** Mohammad Majam, Mothepane Phatsoane, Theodore Wonderlik, Naleni Rhagnath, Laura K. Schmucker, Leanne Singh, Michael Rademeyer, Harsha Thirumurthy, Noora Marcus, Samanta Lalla-Edward

**Affiliations:** ^1^ Ezintsha, Faculty of Health Sciences, University of the Witwatersrand, Johannesburg, South Africa; ^2^ Department of Medical Ethics and Health Policy, Perelman School of Medicine, University of Pennsylvania, Philadelphia PA, United States; ^3^ A2D24, Randburg, South Africa

**Keywords:** HIV care cascade, financial rewards, HIV management, HIV linkage to care, HIV self-test, South Africa

## Abstract

**Introduction:**

Achieving viral suppression in people with HIV is crucial in ending the AIDS epidemic. Among users of HIV self-screening tests, low rates of linkage to care and early retention in care are key obstacles to achieving viral suppression. This study sought to evaluate the efficacy of financial incentives in supporting HIV case management.

**Methods:**

Young adults within the inner city of Johannesburg, South Africa and surrounding areas who used HIV self-tests, were able to use WhatsApp to communicate with study personnel, reported a reactive or invalid result, and were confirmed to by HIV-positive were enrolled in the study. Participants were randomised to an intervention arm that received reminders and financial rewards for engaging in care, or to a control arm that received the standard of care. The primary outcome was HIV viral load at six months.

**Results:**

Among 2,388 HIV self-test kits that were distributed, 1757/2,388 (73,58%) recipients were able to use their phones to send photos to study personnel. 142/1,757 (8,08%) of these recipients reported reactive or invalid results. Upon confirmatory testing, 99/142 (69,71%) participants were identified as being HIV-positive and were enrolled in the study. 2 (1,41%) participants received an HIV negative result, and 41(28,87%) participants were either lost to follow-up or did not complete the confirmatory testing step. 20/99 (20,2%) from the intervention arm and 18/99 (18,18%) from the control arm completed the study (i.e., attended a 6 month follow up and participated in the exit interview). 29/99 (29,29%) were virally suppressed by at 6 months. Of those achieving viral suppression 15 (51,72%) were from the intervention arm.

**Conclusion:**

Financial incentives and reminders were not effective in promoting engagement with HIV care and viral suppression in this setting.

## Introduction

The sustained use of antiretroviral therapy (ART) is crucial for achieving viral suppression among people living with HIV and preventing onward HIV transmission (1). Achieving viral suppression hinges on the prompt identification of individuals living with HIV, and subsequent access to, and sustained retention in HIV care. Thus far, while there has been significant advancement globally in the realisation of the first two “90s” of the WHO's 90-90-90 targets (90% of people living with HIV knowing their status and 90% of HIV patients on ART respectively), achieving the final “90” (viral suppression in all who receive ART) has not been reached (2–4). It has been proposed that to be able to maintain a successful HIV continuum of care and move closer to reaching the final “90” target, timeous testing for HIV is imperative, since no or delayed testing exacerbates infection transmission (5). Ghamie G, et al. emphasise that innovative and periodic HIV testing procedures need to be adopted to reach parts of the population who are unaware of their HIV status (6).

Various studies have been conducted to identify the problems associated with HIV testing and linking into HIV care and to make recommendations on how to bridge the current gaps in the HIV treatment continuum. This is critical particularly in the low and middle income region of Sub-Saharan Africa, as this region significantly lags behind western countries when it comes to patients initiating and sustaining the use of ART (7).

One recommendation is that HIV testing take the form of HIV rapid diagnostic tests (RDT), conducted by patients themselves in the form of HIV self-screening (HIVSS), to facilitate the uptake of HIV infection identification and consequent treatment, as HIVSS has shown to have high acceptability across a wide range of populations. For low- and middle-income settings this is particularly significant, as these settings bear a high burden of HIV infections and previous studies have reported a growth in testing when HIVSS has been used. This is largely because HIVSS alleviates the tester's confidentiality, cost, and convenience concerns (8–10).

However, it has become apparent that the uptake of HIV testing does not necessarily translate into boosting accessing of care, and that further mediation strategies are essential (11). Choko, et al, reiterate this notion and suggest that while there is no one-size-fits-all solution, bespoke mediation strategies can be formulated to address the HIV testing and continued access to care needs of different population groups, from available and emerging care linkage data. For example, in South Africa text messages to confirmed HIV positive patients increased the likelihood of them linking to care (12).

One popular mediatory mechanism that has been proposed is the use of incentives. Stoner, et al, in their 20-year exploration of the impact of disbursing various forms of financial incentives to at risk groups, found that there was an inconsistent link between the disbursement of financial incentives and the prevention of HIV infection (13). Krishnamoorthy, et al, in their examination into whether financial incentives positively impacted the uptake of HIV care, concluded that it does have the potential to improve patient retention in the HIV cascade of care (14). A year-long review of household economic strengthening (HES) strategies as an approach to stimulating uptake in HIV testing and care, drew two significant conclusions. The first is that financial rewards stimulate the uptake of HIV testing and linkage to care in adults generally and in specific contexts like when financial rewards can help people to pay for transportation expenses. The second is that it is difficult to pinpoint trends amongst the various HES schemes given that there are many variables to consider (15).

Another disconcerting reality is that there is also a need for HIVSS and HIV care interventions that target men in particular because they have lower levels of engagement in testing and treatment (16, 17). Whereas women often access testing around childbirth, men access healthcare (including HIV testing) more rarely and as a consequence are diagnosed later in the HIV disease progression (18, 19) and are in addition less likely to link to care than women (20). Identifying interventions to increase HIV testing and linkage to care among men – particularly those engaged in high-risk behaviours remains an HIV prevention priority.

Drawing on health and behavioural economics, this pilot study developed and tested a financial incentive intervention programme to measure the effect of a modest financial incentive offered in: (1) completing a confirmatory HIV test following a positive HIVSS test result, and (2) demonstrating viral suppression by approximately 6-months after a positive HIVSS test result. By testing whether targeted, low-cost incentives for facilitating HIV care access and viral suppression are effective in the context of HIVSS, the results from this study, reported on here, can inform larger-scale efforts, in South Africa and in other countries in the region, in achieving strengthened HIV care cascades.

## Materials and methods

### Study design

This study took place from July to December 2020 and enrolled 142 participants. Upon meeting the inclusion criteria, participants were randomly assigned to intervention or control: the intervention group receiving financial incentives for confirmatory testing, linking to care and viral suppression – or the control group receiving standard of care (SOC) for linking to care.

All participants who completed key study procedures received standard financial compensation for time and transportation.

### Study site

Locations which yielded high numbers of men and young adults within the inner city of Johannesburg, South Africa as well as its surrounding areas of Alexandra, Soweto and Yeoville, were deliberately selected for the distribution of HIV self-test (HIVST) kits. In 2020, the HIV prevalence of the City of Johannesburg was 13% (21). Confirmatory testing of HIV positive results was undertaken at the Ezintsha Research Centre in Hillbrow, Johannesburg, South Africa.

### Study population

Convenience sampling was used to recruit candidates already participating in the following initiatives: STAR HIVSS distribution programme, Sedia, and Hepatitis C virus (HCV) product evaluation studies in operation at the Ezintsha research clinic and ANOVA's harm reduction initiative at the Yeoville clinic. Both males and females were recruited on condition that they were willing to not only test for HIV, but also share their results digitally *via* short messaging service (SMS) or WhatsApp. Race, gender, ethnicity, and sexual orientation were disregarded.

After distributing 2,388 HIVST kits to potential study participants, from the 402 candidates who responded, 142 were eventually deemed eligible for confirmatory testing and possible inclusion in the study. Eventually, 99 participants who had fulfilled all the study criteria comprised the study sample, 49 of whom were assigned to the intervention arm and 50 to the control arm.

### Inclusion and exclusion

Eligible participants were 18 years or older at the time of the study and had reported an HIV positive status after being tested. They also had access to a phone with a personal or valid phone number that was going to be active for at least six-months post HIV self-screening and which had a WhatsApp or text messaging feature. Each participant also needed to have understood and signed the informed consent form.

Ineligible participants were those who did not meet the eligibility criteria, were not willing to undergo a confirmatory test after testing positive for HIV and were unwilling to provide informed consent.

### Recruitment process

Field workers from the STAR HIVSS distribution programme, using either the fixed HIVSS distribution or the door-to-door distribution channel, disseminated an HIVSS kit, IFU pamphlet and a uniquely barcoded result card, to passers-by and residents. Recipients who tested positive for HIV were requested to share their result with the study staff to a designated phone number *via* their mobile phones. This could be done by sending either a picture of their positive results on the results-card (Supplementary Figure S1) *via* WhatsApp or *via* SMS reporting the positive result and quoting the unique barcode from the test kit. These candidates as well as those with confirmed HIV positive results from the Sedia and HCV studies as well as the ANOVA-Drug users harm reduction initiative, who presented themselves for routine testing at the Yeoville site, were provided with the opportunity to participate in the study. They too were required to submit text messages or photographic evidence of their HIV positive results.

### Study procedure

#### Confirmatory testing

After the validation of HIV positive results by study staff, participants were contacted by a linkage officer within three days to conduct an informed consent telephonically before proceeding with randomly grouping participants into the intervention and control. At this stage, only STAR candidates received ZAR50 (approximately USD 3,00) for the photographic evidence of positive results sent *via* WhatsApp. All participants with an HIV positive result were required to undergo confirmatory testing at the Ezintsha research clinic before they could be manually randomized into a particular study arm. All participants who presented themselves for confirmatory testing were reimbursed ZAR150 (approximately USD 9,00) for time and transport costs, with those from the STAR programme who randomised in the intervention arm receiving a further R75 (approximately UDS 4,90) for completing confirmatory testing. The research nurse performed a blood draw for the baseline viral load (VL). All participants were advised to attend an ART clinic of their choice.

#### Randomisation

Participants were randomised into one of two study arms after their HIV positive test results were confirmed, as described above. Random number generation was used for randomisation and Stata for assignment. To facilitate randomisation for Sedia, ANOVA and HCV participants, they were also required to submit images of their self-test RDT for verification to the same telephone number as STAR candidates. Upon randomisation into their specific arm, the letter T was attached to the end of the intervention arm candidate's patient identity number (PID) and the letter C attached to the end of the control arm candidate's PID. An additional code was added to the end of the aforementioned coding to indicate whether or not the patient had access to WhatsApp (M if no WhatsApp). A further code preceded the PID to indicate the group from which the patient was recruited: S for the Sedia group, A for ANOVA group, and H for the HCV group. While no balance tests were run during the randomisation process, participants were randomised 1:1, so that there was an even distribution among the participants from each recruitment site or programme.

#### Follow up visits

With regards to medication pick-up reporting, monthly reminders to collect medication from their nearest clinic were sent to all intervention arm participants *via* text messaging or WhatsApp. Participants were requested to send a picture of their collected medication to the study WhatsApp number. Participants whose WhatsApp pictures were verified by study staff received a ZAR25 (approximately USD 1,50) reward *via* e-Wallet. Incentivisation was not extended to participants without WhatsApp or who did not submit an image.

#### End-line VL

Approximately six months after receiving and verifying the image from the participant indicating an HIV positive reading, up to five follow-up calls (one per week unless an additional call within a particular week was warranted) were made to each candidate inviting them to complete a follow-up visit at the study clinic. Voice messages were left for unreachable participants. On presenting themselves at the clinic, informed consent was administered by the linkage officer before the completion of the exit interview questionnaire and collation of demographic data. Participants subsequently had their blood sample (4 ml EDTA) drawn by a research nurse for laboratory VL PCR testing. These returning participants were reimbursed for their time and transportation cost. Moreover, intervention arm participants with a VL < 400 copies/ml were rewarded further with ZAR300 (approximately USD 18). On completion of all procedural steps, participants were exited from the study once they completed an exit questionnaire.

**Figure 1:**
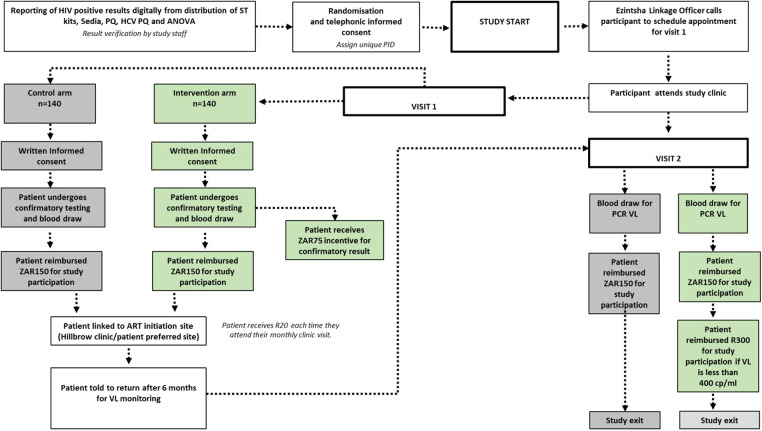
Study design workflow.

**Figure 2:**
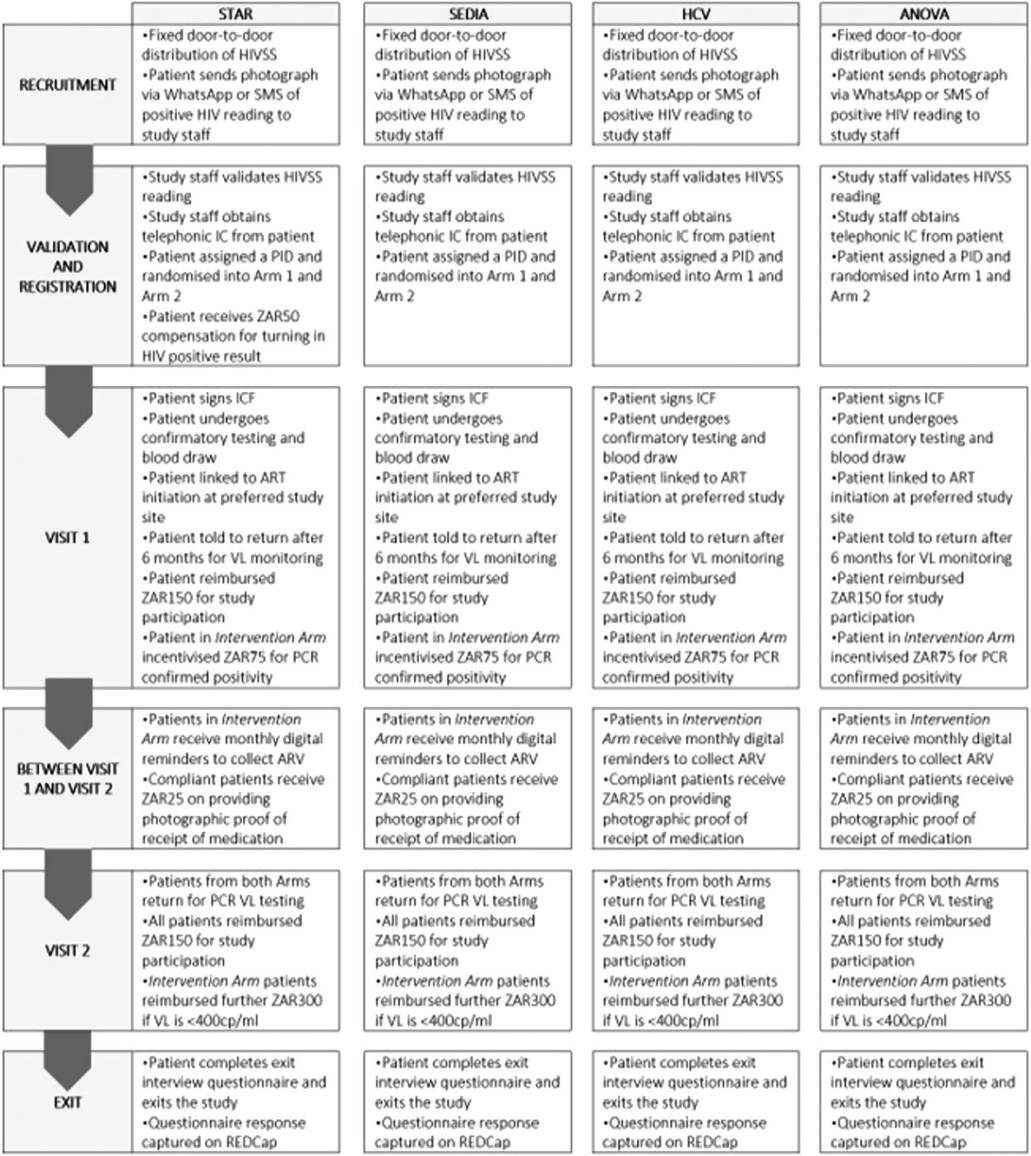
Work study process per recruitment group. HCV, hepatitis C virus; HIVSS, HIV self-screening; SMS, short messaging services; IC, informed consent; PT, participant; PID, patient identification; ICF, informed consent; ART, antiretroviral therapy; VL, viral load; PCR, polymerase chain reaction; ZAR, South African rand; cp/ml, copies per millilitre.

Figures 1,2 show the study design workflow and work study process per recruitment group respectively, while Supplementary Table S1 contains the incentive summary.

### Data management

Data management was performed by the research staff who created the standard operating procedures for maintaining the confidentiality of participants as well as all the data (both paper-based and electronic) and the transfer, entry and storage levels of the data. Only protocol approved (by the ethics committee) study team members had access to the study data and sharing of any study information beyond the boundaries of the approved study team was disallowed. Data were securely stored by Ezintsha for the regulatory authority mandated storage period.

### Data collection

Two methods were used to gather participant demographic and contact details data: (i) manual paper-based collection form and (ii) electronically utilising a phone on the A2D24 Open Data Kit (ODK) platform (Table 1). The word “positive” along with the test kit unique barcode was sent by participants who tested positive and who were enrolled using the manual process, to a number provided by the field-worker. Thereafter, these participants' tests results were confirmed at the Ezintsha Research Clinic before being manually randomised into a particular study arm, the summary of which is contained in Table 1, and pictorially represented in Supplementary Figures S2A–C. Exit interview question responses were captured on REDCap by the designated data-capturer.

**Table 1:** Data collection summary.

Manual (DCF)	Electronic (A2D24 ODK platform)
STAR Programme cell phone without WhatsApp	STAR Programme cell phone with WhatsApp
HCV mobile phone without WhatsApp	HCV mobile phone with WhatsApp
Sedia mobile phone without WhatsApp	Sedia mobile phone with WhatsApp
ANOVA without WhatsApp	ANOVA with WhatsApp

DCF, data collection form; ODK, open data kit; HCV, hepatitis C virus.

### Training

The training of study staff was informed by the study training material. On culmination of the training, competency logs were completed, signed by team members, and filed.

### Ethical considerations

Ethical approval was received from the Human Research Ethics Committee Members of University of the Witwatersrand (Ethics Reference: 191121) and the Research Committee of Johannesburg Health District (DRC Reference: 2020-09-007).

## Results

### Demographics from kit distribution and enrolment

A total of 2,388 HIVST kits were distributed comprising 1090/2,388 (45,64%) male recipients and 1298/2,388 (54,36%) females. Altogether, 1757/2,388 (73,58%) of candidates were able to report their results in a photo of their HIVST results *via* WhatsApp and 631/2,388 (26,42%) could not. 142/2,388 (5,95%) who turned in a positive or invalid result and subsequently verified as such by study staff, comprised 56/142 (39,44%) responses from male candidates and 86/142 (60,56%) from female candidates. 83/142 (58,45%) candidates who were able to send photographs were assigned to the A2D24 workflow, while the balance, 59/142 (41,455%) were assigned to the manual workflow, all 124 being deemed eligible for the study. Eventually, 138/142 (97,18%) of eligible candidates were indeed true positives and qualified for inclusion in the entire study. The participant yields recruited from the various studies are as follows: STAR - 60/138 (43,48%), Sedia study - 64/138 (46,38%), ANOVA clinic – 8/138 (5,8%), and HCV study – 6/138 (4,35%) (Table 2).

**Table 2:** Demographics from kit distribution and enrolment.

	Frequency (*n*)	Percentage (%)^a^
Kits distributed	2 388	
Sex		
Male	1090	45,64%
Female	1298	54,36%
Able to send photo (ASP)		
ASP	1757	73,58%
Not ASP	631	26,42%
HIVSS result reported (positive or invalid)	142	
Sex		
Male	56	39,44%
Female	86	60,56%
Able to send photo (ASP)		
ASP	83	58,45%
Not ASP	59	41,55%
Recruitment/enrolment method (positive)	138	
STAR distribution	60	43,48%
Sedia study	64	46,38%
ANOVA clinic	8	5,80%
HCV study	6	4,35%

^a^ Due to rounding percentages may not always total to 100.

*n*, number; HIVSS, HIV self-screening; ASP, able to send photo; HCV, hepatitis C virus.

### HIVST reporting and randomisation

Of the self-reported results, 138/402 (34,33%) candidates reported a positive reading and 4/402 (1,00%) of the results were invalid, legitimising these candidates for inclusion into the study. 73/142 (51,41%) candidates were randomised into the intervention arm and 69/142 (48,59%) into the control arm. 2/142 (1,41%) participants were confirmed HIV negative, and 41/142 (28,87%) participants had pending HIV results by the time recruitment had closed or had been lost to follow (unreachable after five attempts to contact them). Amongst all the confirmed positives, there were 21 more females (60/99, 60,61%) than males (39/99, 39,39%). Baseline VL results were available for 95/99 (95,96%) participants, and 55/95 (57,89%) had a VL < 400 copies/ml. Most of the participants (60/95, 63,16%) were enrolled from the Sedia study, within the confirmed positive patients group (Tables 3A,B).

**Table 3(A):** HIVST reporting and randomisation.

	Frequency (*n*)	Percentage of subcategory (%)^a^	Percentage of total distribution (%)^a^
Kits distributed	2 388		
HIVSS results reported	402		16,83%
HIVSS positive	138	34,33%	5,78%
HIVSS negative	260	64,68%	10,89%
HIVSS invalid	4	1,00%	0,17%
Randomisation eligibility and confirmatory testing (HIVSS reported positive or invalid)	142	35,32%	5,95%
Study arms			
Intervention arm	73	51,41%	
Control arm	69	48,59%	
Result confirmation			
Confirmed positive	99	69,72%	4,15%
Confirmed negative	2	1,41%	
Confirmation pending at time recruitment closed or lost to follow-up (not reached after 5 attempts)	41	28,87%	

^a^ Due to rounding percentages may not always total to 100.

HIVST, HIV self-test; *N*, number; HIVSS, HIV self-screening.

**Table 3(B):** Confirmed positive distribution.

	Frequency (*n*)	Percentage (%)^a^
Confirmed positive	99	
Baseline VL for those confirmed positive	95	95,95%
VL < 400	55	57,89%
VL > 400	40	42,11%
Randomization for those with a baseline VL		
Intervention arm	47	49,47%
Control arm	48	50,53%
Sex		
Male	36	37,89%
Female	59	62,11%
Able to send photo (ASP)		
ASP	44	46,32%
Not ASP	51	53,68%
Recruitment/enrolment method		
STAR distribution	23	24,21%
Sedia study	60	63,16%
ANOVA Clinic	7	7,37%
HCV study	5	5,26%

^a^ Due to rounding percentages may not always total to 100.

*n*, number; VL, viral load; ASP, able to send photo; HCV, hepatitis C virus.

### Incentives and follow up

Within the intervention arm, 20 confirmed positive participants with a baseline VL were part of the A2D24 workflow, rendering them eligible for the medication pick-up incentive. Three quarters (15/20) of this had a baseline VL < 400 copies/ml, and 5/15 (23,81%) showed a baseline VL > 400 copies/ml. In this group, 13/20 (65,00%) of participants shared at least one example of photographic evidence of having collected their medication. Among these 13 11/13 (84,62%) of whom had a baseline VL < 400 copies/ml and 2/13 (15,38%) displaying a baseline VL > 400 copies/ml (Table 4A).

**Table 4(A):** monthly medication collection incentive.

	Frequency (*n*)	Percentage of subcategory (%)^a^	Percentage of total (%)^a^
Eligible for pick-up incentive	20		
Baseline VL < 400	15	71,43%	
Baseline VL > 400	5	23,81%	
Medication pick-up shared	13		65,00%
Baseline VL < 400	11	84,62%	
Baseline VL > 400	2	15,38%	

^a^ Due to rounding percentages may not always total to 100.

*n*, number; VL, viral load.

All 99 participants who tested HIV positive during the confirmatory testing procedure were entitled to a follow-up visit six months after the commencement of their participation in the study. Less than half of these patients (38/99; 38,38%) attended the follow-up visit, 20/38 (52,63%) of who were from the intervention arm and 18/38 (47,37%) from the control arm. Furthermore, 29/38 (76,32%) participants exhibited an end line VL < 400 copies/ml whereas 9/38 (23,68%) patients had an end line VL > 400copies/ml. Most of this group, (61/99; 61,62%) were either pending or lost to follow-up by the close of study. Although the study team did not believe that the recruited sample size was sufficient to adequately measure a statistically significant effect, we did run a Fischer's exact test for which the *p*-values are presented in the Table 4B.

**Table 4(B):** 6-month follow up.

	Intervention (*n* = 49)		Control (*n* = 50)		
	Frequency	Percentage	Frequency	Percentage	*p*-value
Eligible for 6 month follow-updd					
Completed	20	40.82	18	36.00	0.682
Pending/Lost to care	29	59.18	32	64.00	
Viral load completed at 6 months					
VL < 400	15	75.00	14	77.78	1.000
VL > 400	5	25.00	4	22.22	

*n*, number; VL, viral load.

### Exit interview

34 participants completed the exit interview questionnaire. The majority (26/34, 76,47%) were in the 26–45-year age range. 24/34 with an HIV diagnosis were also in the 26–45 year age range. While 24/34 (70,58%) were newly diagnosed, by the close of the study, majority self-reported that they had already commenced HIV treatment (33/34 (97,05%) and achieved viral suppression (31/34 (91,17%). 32/34 (94,11%) participants reported collecting their medication regularly. 33/34 (97,05%) participants enrolled in the study to gain knowledge of their HIV status, while 13/34 (38,23%) were motivated by the financial incentive (Table 5).

**Table 5:** Exit interview data collection summary.

Criteria		*N* = 34	Percentage (%)^a^
Demographics			
Age			
	18–25	2	5,88%
	26–35d	12	35,29%
	36–45	14	41,18%
	46–55	6	17,65%
Education			
	Less than high school	1	2,94%
	Some high school	22	64,71%
	High school graduate	4	11,76%
	College or specialised training	3	8,83%
	College or university graduate	4	11,76%
Employment			
	Yes	8	23,53%
	No	26	76,47%
HIV diagnosis			
Date of diagnosis			
	New < 2yrs ago	24	70,59%
	2 – 10yrs ago	7	20,59%
	10 + yrs ago	3	8,82%
HIV diagnosis age			
	18–25	5	14,71%
	26–35	13	38,24%
	36–45	11	32,35%
	46–55	5	14,70%
ART			
ARV – current			
	Yes	33	97,06%
	No	1	2,94%
ARV – initiation			
	New < 2 yrs ago	27	79,41%
	2–10 yrs ago	3	8,82%
	10 + yrs ago	3	8,82%
	Blank	1	2,94%
Pick-up meds frequency			
	Monthly	8	23,53%
	Every 2 months	12	35,29%
	Every 3 months	11	32,35%
	Every 4 months	1	2,94%
	Other (incl. blank)	2	5,88%
Viral suppression goal			
	Yes	32	94,11%
	No	2	5,88%
Achievement of suppression goal			
	Yes	31	91,18%
	No	1	2,94%
	Not answered	2	5,88
Motivators for study participation			
Knowledge of HIV status			
	Strongly agree	18	52,94%
	Agree	15	44,12%
	Strongly disagree	1	2,94%
Money			
	Strongly agree	6	17,65%
	Agree	7	20,58%
	Neither agree not disagree	2	5,88%
	Disagree	8	23,53%
	Strongly disagree	11	32,35%
Study feedback			
Satisfaction with study experience			
	Very much	29	85,29%
	Neutral	3	8,82%
	Fairly	2	5,88%

^a^ Due to rounding percentages may not always total to 100.

*n*, number; yrs, years; incl, including.

## Discussion

Meeting all the World Health Organization's 90-90-90 goals remains elusive in sub-Saharan Africa. Irrespective of whether HIV testing is home based or community location based, a recent study asserts that the location of the testing has no significant impact on the extent to which HIV positive patients access HIV care, although patients most likely to seek care further are previously diagnosed as opposed to newly diagnosed ones (22). Furthermore, attracting males not just for HIV testing but for continuous ART remains unsatisfactory, and even incentives cannot guarantee male engagement (23). Even newer innovative approaches like mobile health (mHealth) technology while effective in communicating reminders to HIV positive patients, still experienced efficacy impediments due to illiteracy, for example (24).

The assertions are significant in the light of our study. At the outset, despite a deliberate attempt to recruit at least an equal number of males and females for participation in the investigation, fewer males were reached and a significantly lower number of them formed part of the study sample. Whether this is because the number of males who tested positive was much lower than their female counterparts, or whether the male respondents number is lower because of apathy on their part is unclear. If the reason is the latter, it supports the trend that was expounded on in a previous South African study where across the HIV treatment continuum, females have proved to be more responsive (25). Sileo et. al. concluded in their study, that “masculine norms” like stigma concerns and inaccurate assumptions of the effect of HIV treatment negatively affected male engagement (26). Consequently, it is also plausible that this could have been a driving factor for the lower male responsiveness.

Using financial incentives in the identification and management of HIV in sub Saharan Africa appears to have the potential to positively impact adolescents, a recent study shows (27). Other studies undertaken in rural Uganda showed that while male engagement is directly proportional to the value of the “prize” or incentive, incentives do have the potential to increase male participation generally (28) and a separate randomised trial indicated that financial incentives had no impact on the commitment to viral suppression in HIV positive individuals (29). The idea of rewarding individuals financially was not met with enthusiasm in a recent investigation in Cape Town, South Africa either. It emerged that incentivisation received a lukewarm reception from some patients as well as healthcare workers, on the grounds of morality (30). That financial incentivisation, irrespective of the form or monetary value cannot be relied upon to increase engagement with the HIV management continuum amongst adults and particularly male ones has been reinforced in our study too, as the poor responsiveness despite the promise of incentivisation, pointed to it being ineffectual.

It appears then that the action of incentivising HIV testing and adherence to treatment needs to be administered as a complement to additional strategies, like HIV and social responsibility educational programmes, for it to be taken seriously as a factor in promoting responsible treatment behaviour in HIV patients and the public at large.

### Limitations

COVID-19 lockdowns adversely affected recruitment, participant follow-up, and measurement of viral loads at 6 months. The study workflow required participants to have WhatsApp for results and medication picture sharing. This was challenging as many potential participants did not have smartphones, necessitating the introduction of a parallel workflow for them. Subsequently contact number verification became a challenge resulting in participants providing incorrect phone numbers which led in part to the high rate of participants who did not complete follow-up. Whether incorrect numbers were offered deliberately (and if so, why) or in error is unclear. Social status was not used to determine study eligibility which could have altered the study outcome (e.g. with respect to types of phones, and study completion rates). Using convenience sampling may have introduced a bias in that participants joining from other research studies may have been more inclined to have health seeking behaviour. Lastly, as no other modes of communication (e.g., email) were explored this could have also affected the completion rates.

## Conclusion

In this pilot trial, we did not find evidence that financial incentives over and above the reimbursement provided for time and travel are effective in increasing engagement with the HIV care continuum among people living with HIV. This can be seen in the relatively even number of participants who completed follow-up between study arms (20 intervention vs. 18 control). Several of these participants mentioned being motivated by a desire to take control of their health rather than the financial incentives.

## Data Availability

The raw data supporting the conclusions of this article will be made available by the authors, without undue reservation.
