# Supplementary material for: Incentives to promote accessing HIV care and viral suppression among HIV self-screening test users who obtain a reactive result
Source: Front Reprod Health. 2022 Oct 3;4:976021. doi: 10.3389/frph.2022.976021 (PMC9580778; doi:10.3389/frph.2022.976021)
Supplement: Supplementary file 1 [file Presentation1.pptx]

## Slide 1
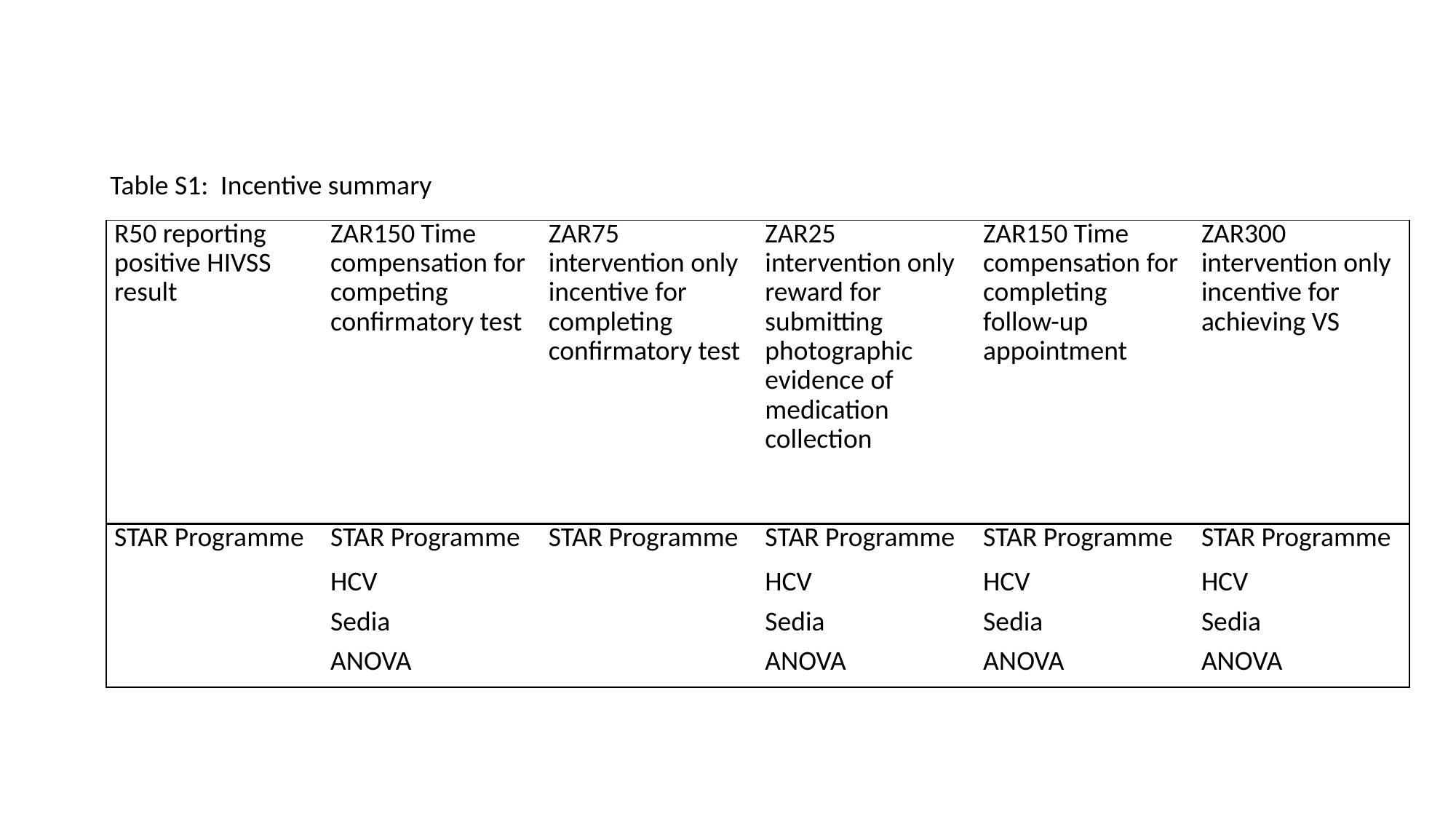

Table S1: Incentive summary
| R50 reporting positive HIVSS result | ZAR150 Time compensation for competing confirmatory test | ZAR75 intervention only incentive for completing confirmatory test | ZAR25 intervention only reward for submitting photographic evidence of medication collection | ZAR150 Time compensation for completing follow-up appointment | ZAR300 intervention only incentive for achieving VS |
| --- | --- | --- | --- | --- | --- |
| STAR Programme | STAR Programme | STAR Programme | STAR Programme | STAR Programme | STAR Programme |
| | HCV | | HCV | HCV | HCV |
| | Sedia | | Sedia | Sedia | Sedia |
| | ANOVA | | ANOVA | ANOVA | ANOVA |
