# Supplementary figures and images for: Incentives to promote accessing HIV care and viral suppression among HIV self-screening test users who obtain a reactive result
Source: Front Reprod Health. 2022 Oct 3;4:976021. doi: 10.3389/frph.2022.976021 (PMC9580778; doi:10.3389/frph.2022.976021)

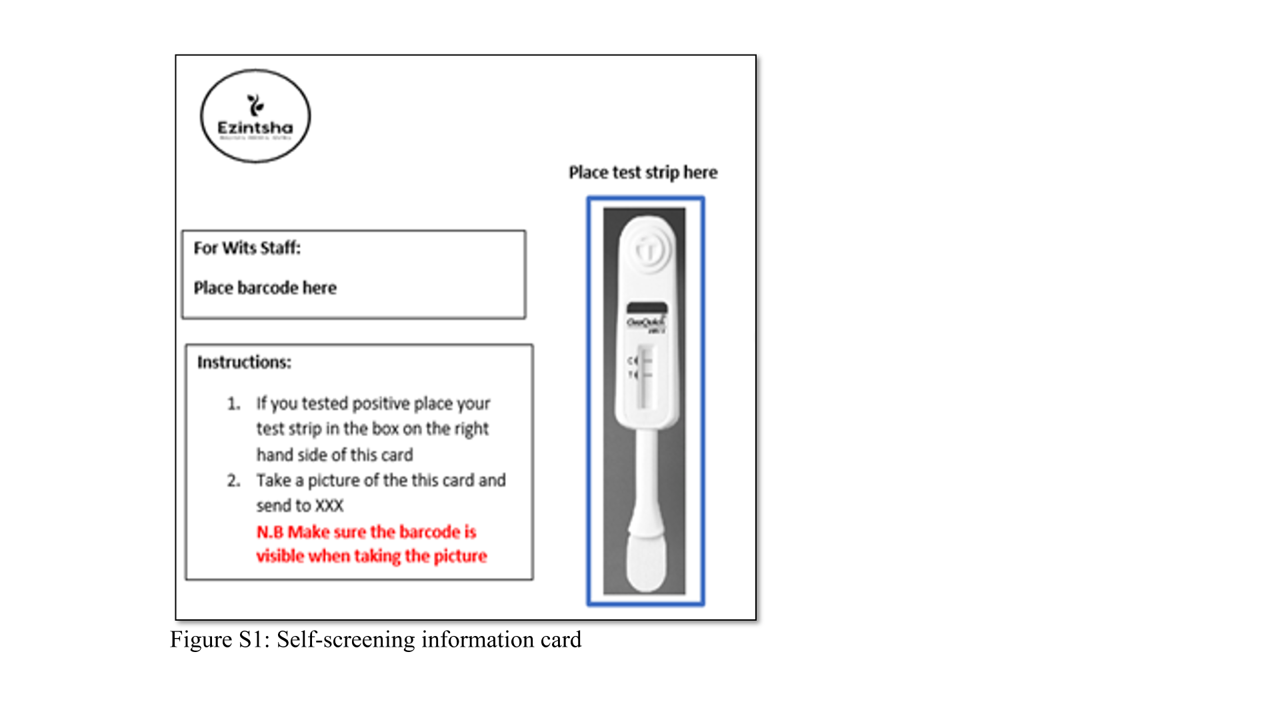

Supplement: Supplementary file 2 [file Image1.tif]

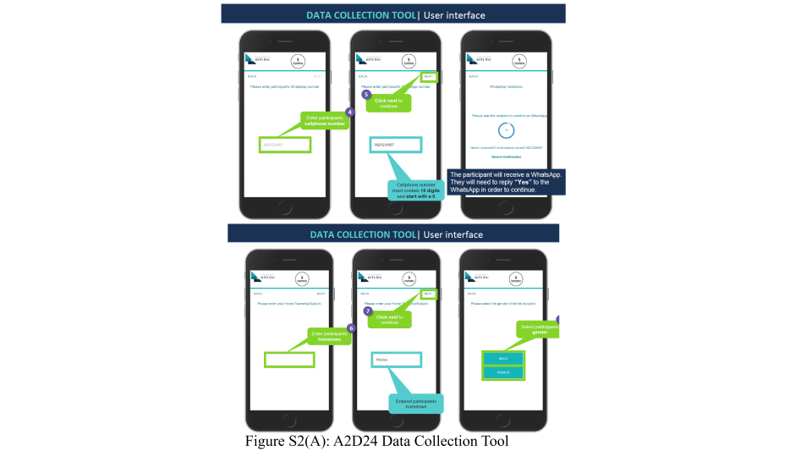

Supplement: Supplementary file 3 [file Image2.tif]

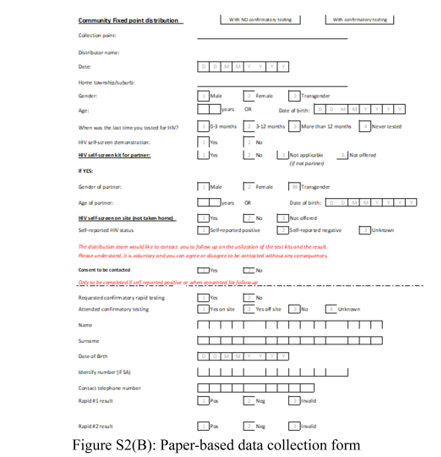

Supplement: Supplementary file 4 [file Image3.tif]

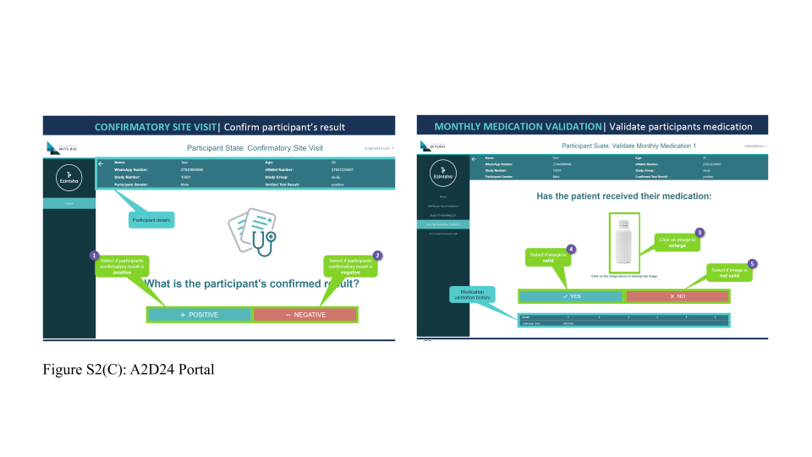

Supplement: Supplementary file 5 [file Image4.tif]
